# Multidrug-resistant Tuberculosis Detection, Latvia

**DOI:** 10.3201/eid1109.041236

**Published:** 2005-09

**Authors:** Girts Skenders, Alicia M. Fry, Inga Prokopovica, Silvija Greckoseja, Lonija Broka, Beverly Metchock, Timothy H. Holtz, Charles D. Wells, Vaira Leimane

**Affiliations:** * State Centre of Tuberculosis and Lung Diseases, Riga, Latvia;; † Centers for Disease Control and Prevention, Atlanta, Georgia, USA

**Keywords:** TB/HIV control, developing countries, multidrug-resistant TB, tuberculosis, TB diagnosis, dispatch

## Abstract

To improve multidrug-resistant tuberculosis (MDR-TB) detection, we successfully introduced the *rpoB* gene mutation line probe assay into the national laboratory in Latvia, a country with epidemic MDR-TB. The assay detected rifampin resistance with 91% sensitivity and 96% specificity within 1 to 5 days (vs. 12–47 days for BACTEC).

Until recent years, global efforts to reduce the prevalence of multidrug-resistant tuberculosis (MDR-TB), defined as in vitro resistance to at least rifampin and isoniazid, have focused on preventing new cases of acquired MDR-TB. However, countries that already have a high incidence of MDR-TB must implement additional strategies, such as reducing transmission by detecting cases earlier and improving infection control in settings with shared air spaces. As yet undetermined are optimal methods to identify drug-resistant *Mycobacterium tuberculosis* in a timely and affordable way in resource-limited settings. Standard laboratory methods of detecting drug resistance, such as *M. tuberculosis* culture and drug susceptibility testing (DST) performed with Löwenstein-Jensen (LJ) medium, are inexpensive but slow; DST results are often not available for 3 to 4 months. Testing methods that use liquid media, such as BACTEC systems (Becton Dickinson, Sparks, MD, USA), can deliver DST results to clinicians within 3 to 4 weeks; however, this technology requires expensive equipment and media.

Several methods that work directly on respiratory specimens and that detect resistance to a limited number of drugs within 1 day to 3 weeks have been reported (*1**–**5*). One assay that is commercially available is a line probe assay, a reverse-hybridization assay that detects mutations in the *rpoB* gene (*5**–**12*). Among clinical *M. tuberculosis* isolates, those with mutations in the *rpoB* gene are associated with 80% to 90% rifampin resistance (*5*). Previously published studies using this assay have demonstrated 90%–100% concordance when results are compared to DST results among *M. tuberculosis* isolates from culture and 78%–98% sensitivity and 84%–100% specificity when applied to respiratory specimens that were positive for acid-fast bacilli (AFB) (*5**–**12*). However, these studies involved small numbers of respiratory specimens and were not performed in a national TB laboratory that supports diagnosis, treatment, and care for large numbers of MDR-TB patients.

Latvia is among those countries with the highest prevalence of MDR-TB in the world (*13*). Rifampin resistance in Latvia is closely associated with resistance to isoniazid; therefore, detecting rifampin resistance should also detect most MDR-TB cases (*13*). As part of a long-term project to integrate new assays into the Latvian national laboratory protocols to identify MDR-TB patients more quickly, we prospectively compared the results of the line probe assay for *rpoB* mutations to results with BACTEC DST technology.

## The Study

We enrolled consecutive patients who were initially seen at or referred to the Latvian State Centre of Tuberculosis and Lung Diseases from January 2003 to March 2004 with AFB-positive respiratory specimens (sputum or bronchoalveolar lavage [BAL] specimens) and identified as being at high risk for MDR-TB. Patients at high risk were defined as those with a history of close contact to a known MDR-TB patient or with a history of previous TB treatment (*14*).

After sputum specimens were decontaminated (*15*), we tested for AFB (*15*) and set up 2 cultures for *M. tuberculosis*: 1 in LJ medium (*15*) and 1 in either the BACTEC Mycobacteria Growth Indicator Tube 960 or the BACTEC 460 system per manufacturer's instructions (Becton Dickinson). DNA was extracted from the remaining suspension with the QIAAMP DNA Mini kit (Qiagen, Valencia, CA, USA). Lysate was transferred to the line probe kit INNO-LiPA Rif.TB (Innogenetics, Ghent, Belgium) for amplification, including a second nested reaction with inner primers and the hybridization reaction (manufacturer's instructions). In general, the *rpoB* gene amplicons were incubated with immobilized, membrane-bound *rpoB* gene probes, including overlapping wildtype sequences (S1–S5) and 4 of the most frequent mutations (R2:Asp516Val, R4a:His526Tyr, R4b:His526Asp, and R5:Ser531Leu). The kit also includes a probe for *M. tuberculosis* complex.

DST was performed with the BACTEC 460 system (manufacturer's protocols). We then compared line probe results to *M. tuberculosis* culture and BACTEC DST results for each patient. We also set up DST on LJ media by using the proportion method (*15*). All laboratory testing was performed at the Latvian State Centre of Tuberculosis and Lung Diseases, Riga, Latvia. Line probe results were not provided to physicians. Patient identifiers were removed before analysis. The protocol underwent institutional ethical review by the Latvian State Centre of Tuberculosis and Lung Diseases, was determined not to be human subjects research, and was approved as programmatic evaluation by the Centers for Disease Control and Prevention.

In total, 89 (37%) of 243 patients who met the case definition for being at high risk for MDR-TB had AFB-positive respiratory specimens; 77 (87%) were sputum specimens, and 12 (13%) were BAL specimens. *M. tuberculosis* isolates grew in BACTEC cultures from 86 (97%) of the AFB-positive specimens. Mycobacteria other than *M. tuberculosis* were identified in 3 of the remaining BACTEC cultures. No dual infections were found. *M. tuberculosis* complex was also detected by line probe assay in 86 specimens, although for 2 patients *M. tuberculosis* grew in respiratory specimens in BACTEC cultures but was not detected by the line probe assay, and 2 specimens that were positive for *M. tuberculosis* complex by line probe assay did not grow in BACTEC but did grow on LJ media. These isolates were injected into the BACTEC 460 system for DST. The line probe assay correctly separated *M. tuberculosis* complex and nontuberculosis mycobacteria.

The line probe assay had good sensitivity, high specificity, and positive predictive value and negative predictive value for rifampin resistance compared to BACTEC (Table 1). Among the isolates resistant to rifampin by BACTEC DST, the *rpoB* mutations detected by the line probe included 20 (61%) R5 (Ser531Leu), 8 (24%) R2 (Asp516Val), 1 (3%) R4b (His526Asp), and 1 (3%) ΔS5 (absence of hybridization to 1 wildtype sequence). One rifampin BACTEC DST-susceptible isolate had a line probe result read as ΔS1, ΔS2 (absence of hybridization to 2 wildtype sequences).

**Table 1:** Comparison of results from line probe assay for *rpoB* gene mutations to rifampin susceptibility results on acid-fast bacilli–positive respiratory specimens*†

Line probe *rpoB* gene mutation results	BACTEC 460 System	
	Rifampin-resistant	Rifampin-susceptible
Resistant	31	1
Susceptible	2	52
No amplification	1	1

*N = 88; Includes 86 *Mycobacterium tuberculosis* isolates from BACTEC plus 2 isolates that grew on Löwenstein-Jensen media. †Compared to results from BACTEC drug susceptibility testing, the line probe assay had a sensitivity of 91% (95% confidence interval [CI] 83–99) and a specificity of 96% (95% CI 92–100). The positive predictive value of the line probe *rpoB* mutation result for rifampin resistance was 94% (95% CI 88–100), and the negative predictive value was 96% (95% CI 92–100).

Most patients considered high risk for MDR-TB had resistance to at least 1 drug (Table 2). Rifampin resistance was highly correlated with classification as MDR-TB; 32 (97%) of 33 patients with rifampin resistance had MDR-TB. The predictive value of the line probe *rpoB* mutation result for MDR-TB was 91% (95% confidence interval 92–100).

**Table 2:** Drug-susceptibility profiles for patients at high risk for MDR-TB with acid-fast bacilli–positive respiratory specimens*†

Drug resistance	No. (%)
None	35 (40.0)
Any resistance	52 (60.0)
Rifampin (total)	34 (38.6)
Mono-rifampin	1 (1.1)
MDR (total)	33 (37.5)
R, H	2 (2.3)
R, H, S	17 (19.3)
R, H, S, E	14 (15.9)
Isoniazid (total)	52 (59)
Mono-isoniazid	5 (5.7)
H, S	14 (15.9)

*N = 88; includes 86 *Mycobacterium tuberculosis* isolates from BACTEC plus 2 isolates that grew on Löwenstein-Jensen media. †R, rifampin; H, isoniazid; S, streptomycin; E, ethambutol; MDR-TB, multidrug-resistant tuberculosis.

The line probe assay performed directly on DNA extracted from respiratory specimens gave quicker results for rifampin resistance (median = 4 days, range 1–5) than other methods (BACTEC 460 median = 28 days, range 12–47; LJ median = 58 days, range 47–65). While DST results from the BACTEC liquid culture system were available considerably faster than were results from LJ media, *rpoB* gene mutation results were available in <1 week.

## Conclusions

In Latvia, where nearly 40% of patients had a history of TB treatment and 10% of all new patients without a history of treatment have MDR-TB (*13**,**14*), integrating a line probe assay for *rpoB* gene mutations into regular laboratory services could enhance MDR-TB control efforts. Results from this study demonstrated that in persons considered at high risk for MDR-TB, the line probe assay detected rifampin resistance with 91% sensitivity and 97% specificity on respiratory specimens within 1 to 5 days of specimen collection in a busy clinical laboratory. Additionally, 91% of patients at high risk for MDR-TB, with line probe assay results consistent with *rpoB* mutations, were ultimately confirmed as having MDR-TB.

In addition, we compared the timeliness of acquiring rifampin DST results between a liquid and solid media system and a line probe assay. Although liquid media were considerably faster than solid media, the line probe assay for *rpoB* mutations performed directly on respiratory specimens gave results consistent with MDR in <1 week.

Therefore, integrating the use of the line probe assay on AFB-positive respiratory specimens into the Latvian national laboratory could permit much earlier segregation and isolation of infectious patients who have a high likelihood of MDR-TB (thereby reducing MDR-TB transmission) and could facilitate more focused DST practices for first- and second-line TB drugs and more efficient use of resources. The high specificity is reassuring; the use of line probe assay results to inform drug treatment selections would rarely result in missed opportunities to treat with rifampin. Conversely, only 9% of patients infected with a rifampin-resistant isolate would not benefit from early detection of resistance and would, in turn, receive care similar to the current standard.

Several other assays that detect drug resistance within 1 to 3 weeks have been described (*1**–**5*). Some of these may perform as well as the line probe assay and be less expensive. We chose the line probe assay for our project because it was commercially available and had been evaluated by several investigators (*5**–**12*). Also, the equipment and skills could be applied toward other molecular epidemiologic studies to better understand ongoing transmission of MDR-TB in Latvia. We will evaluate the cost-effectiveness of integrating this assay into the Latvian State Centre of Tuberculosis and Lung Diseases and may also model the cost of new assays as they become available. This study, part of a larger project to reduce the prevalence of MDR-TB in Latvia, is a first step in identifying optimal methods to identify drug-resistant *M. tuberculosis* in a timely and affordable way in resource-limited settings with high MDR-TB prevalence.
